# A case of gossypiboma diagnosed with transanal double-balloon enteroscopy

**DOI:** 10.1007/s12328-018-0920-y

**Published:** 2018-11-19

**Authors:** Takehiro Ishii, Satohiro Matsumoto, Hiroyuki Miyatani, Hirosato Mashima

**Affiliations:** Department of Gastroenterology, Saitama Medical Center, Jichi Medical University, 1-847 Amanuma, Omiya, Saitama, 330-8503 Japan

**Keywords:** Gossypiboma, Double-balloon enteroscopy, Pelvic mass

## Abstract

Gossypiboma is an iatrogenic granuloma caused by retained surgical gauze. A 48-year-old woman with a history of cesarean section was incidentally found to have a pelvic mass on preoperative computed tomography examination for pectus excavatum. Abdominal enhanced computed tomography showed a 40-mm mass containing air in the pelvis. The mass was suspected to be continuous with the ileum. Transanal double-balloon enteroscopy showed a small fistula that was likely caused by penetration of the ileum dozens of centimeters from the ileocecal valve. A yellow–brown, movable, and fibrous body was found in the fistula. A part of the fibrous body was extracted with forceps. Pathological examination revealed that it was gauze. This is the first reported case of an asymptomatic gossypiboma penetrating the ileum that was diagnosed with double-balloon enteroscopy. Our results suggest that double-balloon enteroscopy is useful for early diagnosis of pelvic mass penetrating intestine, including gossypiboma.

## Introduction

Gossypiboma is an iatrogenic granuloma caused by retained surgical gauze. The term gossypiboma originates from the Latin word “gossypium”, which means “cotton”, and the Swahiri word “boma”, which means “place of concealment [1]”. The term is also reported to be derived from the association with gossip about surgeons. The incidence of gossypiboma has been decreasing owing to technical advances, including the use of radiopaque sutures, and the recently reported incidence is 1 in 5000–10,000 [2]. It is expected that most chronic patients remain asymptomatic for an extended period. Herein, we report a case of gossypiboma diagnosed with transanal double-balloon enteroscopy in a patient presenting with an asymptomatic pelvic mass.

## Case report

**Fig. 1:**
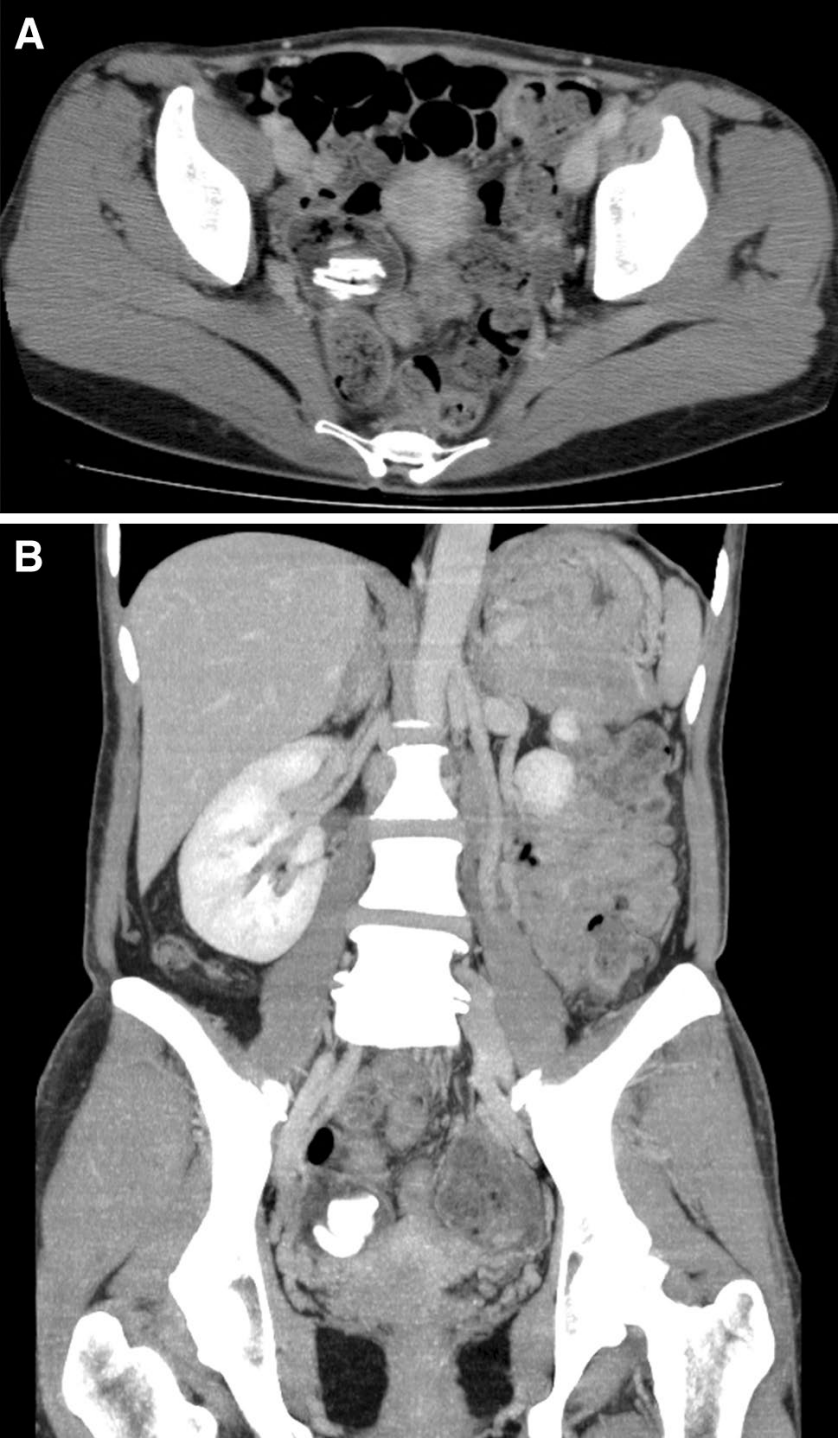
Abdominal enhanced CT showing a 40-mm mass containing air and linear high-density areas. The capsule of the mass had a slight enhancement effect. **a** Axial image, **b** coronal image

**Fig. 2:**
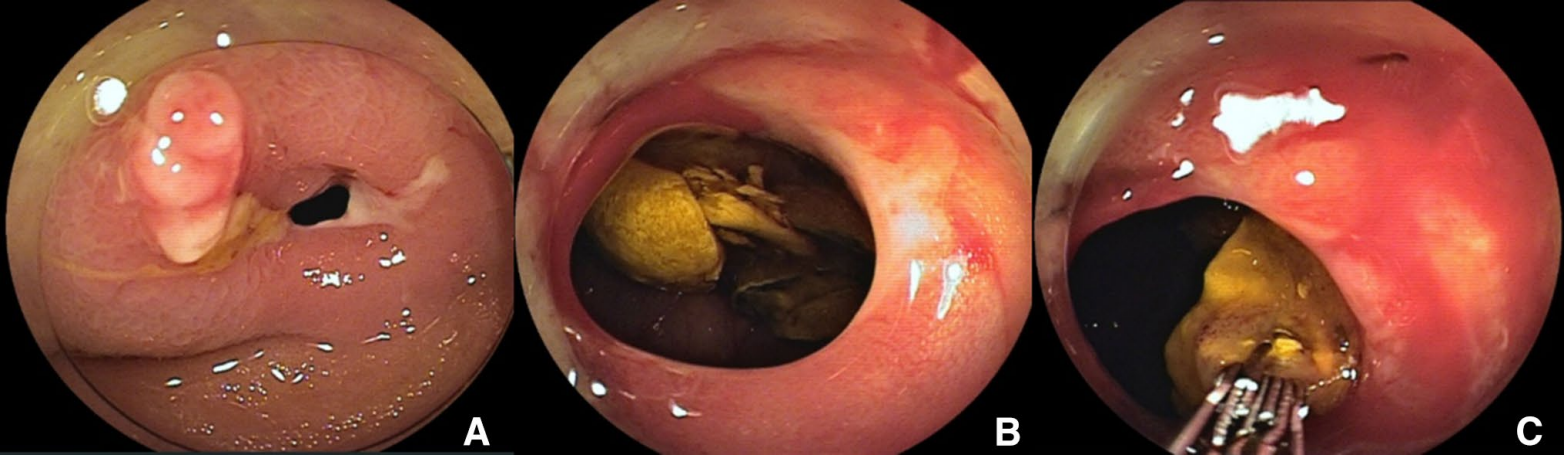
**a** The enteroscopic examination showed a small fistula that was likely caused by penetration of the ileum dozens of centimeters from the ileocecal valve. **b** A yellow–brown, movable, and fibrous body was found in the fistula. **c** The body was held, and part of it was extracted with forceps

**Fig. 3:**
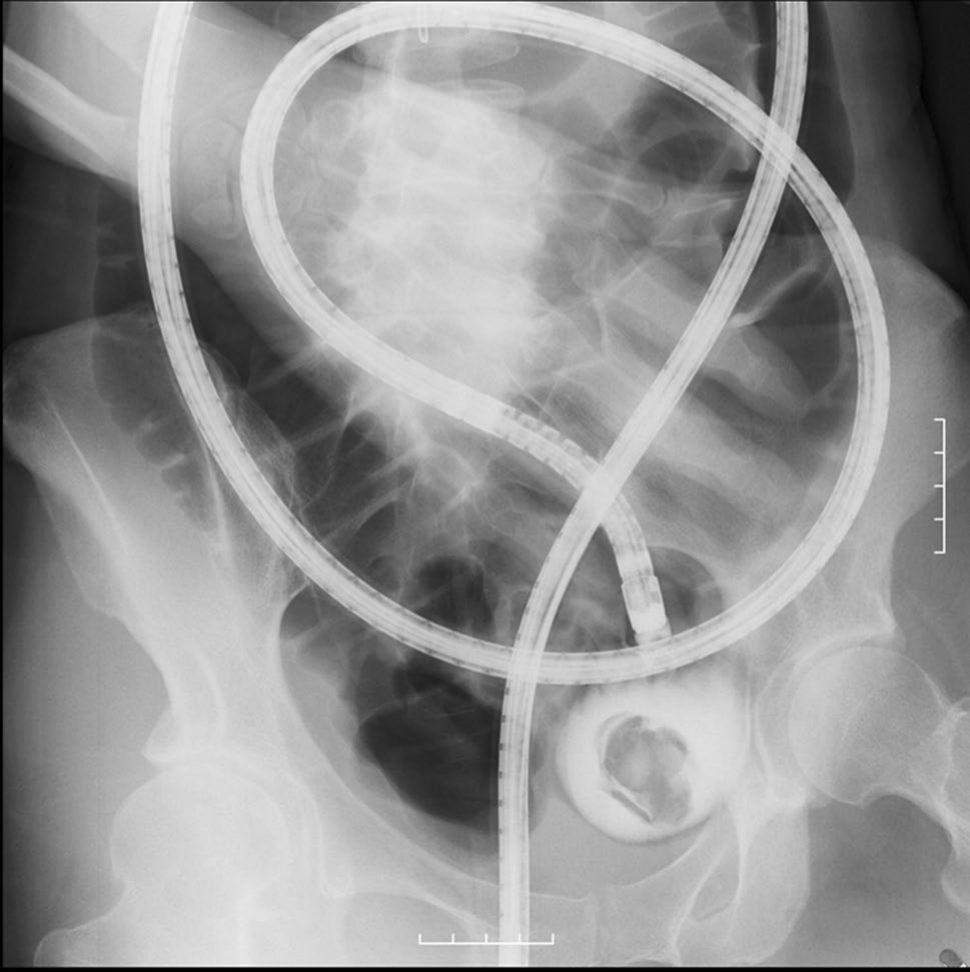
Contrast enhancement via the fistula showed a defect in the enclosed cavity

**Fig. 4:**
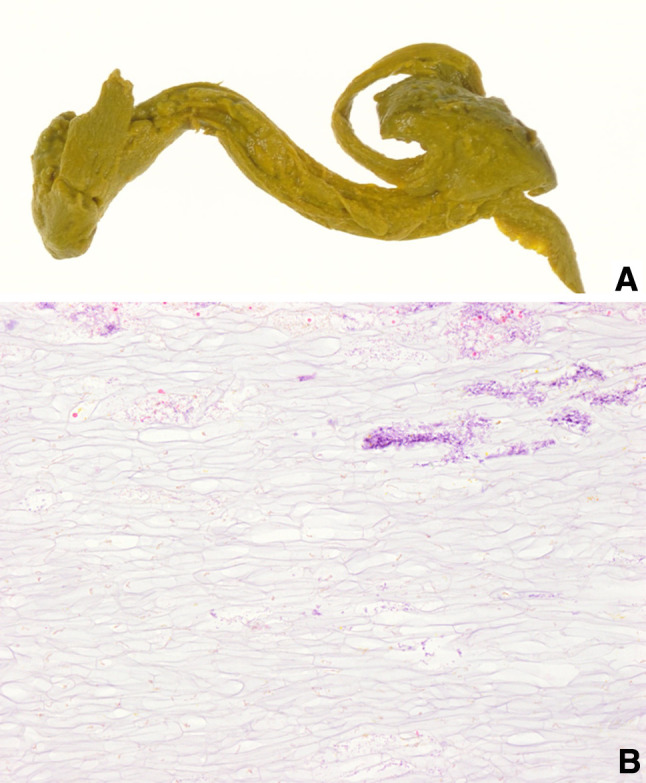
**a** The macroscopic findings: yellow–brown fibrous body. **b** The microscopic findings: foreign body in a reticular pattern

The patient was a 48-year-old woman who had undergone a cesarean section at the age of 35 years. She underwent plain computed tomography (CT) as a screening before surgery for pectus excavatum, and was incidentally found to have a pelvic mass. She was examined by a local gynecologist but showed no remarkable findings and was then referred to our hospital for further investigation of the pelvic tumor. Her height was 169.0 cm; body weight, 52.7 kg; and BMI, 18.5 kg/m^2^. The white blood cell count was 3600/μL; hemoglobin level, 13.5 g/dL; and CRP level, 0.02 mg/dL. A blood test for tumor markers including carcinoembryonic antigen, CA19-9, and CA125 showed negative results. Abdominal plain radiography showed no remarkable findings. Abdominal enhanced CT (Fig. 1) showed a 40-mm mass containing linear high-density areas. The capsule of the mass had a slight enhancement effect. It was not clear whether the mass was continuous with the intestinal tract. However, the mass contained air, thus raising the possibility that the mass was continuous with the intestinal tract. We, therefore, decided to perform transanal double-balloon enteroscopy for further investigation. The enteroscopic examination showed a small fistula that was likely caused by penetration of the ileum dozens of centimeters from the ileocecal valve (Fig. 2a). A yellow–brown, movable, and fibrous body was found in the fistula (Fig. 2b). Contrast enhancement via the fistula showed a defect in the enclosed cavity (Fig. 3). When the body was grasped with forceps, the defect inside was found to be movable. The body was held, and part of it was extracted with forceps (Fig. 2c) and submitted for pathological examination (Fig. 4). The examination of the extracted fibrous body suggested that it was gauze. On the basis these findings, the condition of the patient was diagnosed as gossypiboma penetrating the ileum. Presumably, the gauze had been left during the cesarean section because the patient had not undergone any other abdominal surgery. The diagnosis was explained to the patient and her family. The patient desired to undergo surgery for gossypiboma at the hospital where she had undergone the cesarean section, and was referred to that hospital.

## Discussion

Retained surgical gauze induces two types of responses in the body: exudative and aseptic fibrinous responses. Exudative response is characterized by fever and pain in the early postoperative period and progression to a serious condition with formation of abscess and fistula. Aseptic fibrinous response is characterized by adhesion to the surrounding tissue (without causing inflammation), encapsulation, organization, and subsequent formation of a granuloma [3]. An aseptic fibrinous response is often asymptomatic for an extended period, and the lapse of time between the causal surgery and the discovery of the material ranges from several months to several decades [4]. The clinical symptoms of the present patient were classified as aseptic fibrinous response because she had been asymptomatic for 13 years. In general, imaging studies are useful for the diagnosis. The characteristic findings include a whirl-like appearance on abdominal plain radiography and a whirl-like spongiform pattern on CT, representing gas trapped in the fibers of the gauze; however, these signs are rarely observed [5]. In the present case, we were able to suspect gossypiboma based on the past history of cesarean section and the imaging finding, but could not rule out the possibility of a pelvic tumor with calcification. Although we could not confirm that the mass was continuous with the intestinal tract, we suspected that it was because the mass contained air. Therefore, we performed double-balloon enteroscopy, and a diagnosis of gossypiboma penetrating the ileum was obtained. It is very rare that an iatrogenic granuloma formed by an intra-abdominal foreign body, including gossypiboma, penetrates and migrates into the intestinal tract [4]. Most patients with this condition have natural passage from the anal canal or present with symptoms such as fever, abdominal pain, ileus, and gastrointestinal bleeding, which lead to diagnosis and surgery. There is a reported case of gossypiboma penetrating the large bowel and forming a diverticulum-like lesion and was diagnosed with lower gastrointestinal endoscopy performed for the investigation of anemia [6]. However, to our knowledge, there are no reported cases of an asymptomatic gossypiboma penetrating the ileum that was diagnosed with double-balloon endoscopy.

She was then referred to the university hospital, which belonged to the same group of hospitals as the one in which she had undergone a caesarean section. She underwent surgery at the university hospital to remove the retained gauze. We tried to obtain the surgical records and pathology reports to gain information about the nature of the lesion. However, these data were not made available due to medical concerns. Gawenda et al. found that the frequency of leaving instruments and foreign bodies in the abdomen ranged from 1 in 8801 operations to 1 in 18,760 operations [7]. However, this rate was calculated solely on the basis of malpractice claims. The true incidence may be higher because of the reluctance to report such incidents due to legal and medical issues. Risk factors of gossypiboma were emergency surgery, unplanned changes in the surgical procedure, high body-mass index, duration and complexity of surgery, change in operating room teams during the course of the operation, and a failure to count surgical sponges [7, 8]. All aforementioned conditions did not hold true for the patient, except for the last one since data on the sponge count during the patient’s operation were not available. Currently, surgical gauzes in use are labeled with radiopaque markers. Therefore, retained gauze can be easily identified by radiographic examination [9]. In our hospital, the sponges are counted three times during every operation, while radiographic screening is performed if there is a doubt about the sponge count. When we analyzed the count discrepancy data present in a web-based incident reporting system over a 5-year period (January 2014–September 2018), we found that count discrepancies occurred in 34 of the total 34,034 operations (0.10%). Following careful examination of the peritoneal and/or pleural cavity, surgical gauze was retrieved in 26 operations. With additional radiographic screening, gauze was retrieved in the remaining eight operations as well, thus, the incidence of retained gauze became zero. However, it will take several decades to confirm it.

In the present case, we were able to diagnose the pelvic mass as a gossypiboma penetrating the ileum, using double-balloon enteroscopy, when it was still asymptomatic. Our results suggest that double-balloon enteroscopy is useful for early diagnosis of pelvic foreign-body granuloma penetrating intestine, including gossypiboma in patients with a history of abdominal surgery and presenting with a pelvic mass whose imaging diagnosis was difficult.
